# Adiposity–Metabolic Discordance Identifies Cardiometabolically Heterogeneous Profiles in Normal-Weight Adults: A Cross-Sectional Study

**DOI:** 10.3390/medsci14050557

**Published:** 2026-09-09

**Authors:** Diego González Carrasco, Pedro Juan Tárraga López, Mónica Silu Piña Dabreu, Lluis Rodas Cañellas, Ángel Arturo López-González, José Ignacio Ramírez-Manent

**Affiliations:** 1 ADEMA University School, University of the Balearic Islands, 07009 Palma, Spain; 2 Faculty of Medicine, University of Castilla La Mancha (UCLM), 02008 Albacete, Spain; pjtarraga@sescam.jccm.es; 3 Balearic Islands Health Service, 07010 Palma, Spain

**Keywords:** body mass index, visceral fat, cardiometabolic risk, life style, occupational health

## Abstract

Background: Body mass index (BMI) is widely used to classify obesity and cardiometabolic risk, although it may fail to detect hidden metabolic impairment in individuals with normal body weight. Increasing evidence suggests that adiposity and metabolic dysfunction may not always coexist, giving rise to biologically discordant phenotypes associated with different cardiometabolic profiles. Methods: This cross-sectional study was a secondary analysis of a subset of normal-weight adults selected from a larger database of Spanish workers undergoing routine occupational health examinations between 2019 and 2024. The final analytical sample included 166,008 workers aged 18–69 years with BMI values between 18.5 and 24.9 kg/m^2^. Participants were classified into four adiposity–metabolic phenotypes according to the presence or absence of increased adiposity, operationally defined as a waist-to-height ratio (WtHR) ≥ 0.50, and metabolic dysfunction, defined as the presence of ≥2 of five adverse cardiometabolic criteria. METS-VF was additionally evaluated as a marker of visceral adiposity. Anthropometric, metabolic, vascular, and lifestyle-related variables were analyzed. Multivariable logistic regression models were used to evaluate factors associated with discordant phenotypes. Results: The concordant healthy phenotype was the most prevalent subgroup (78.9%), followed by adiposity-only (9.8%), metabolic-only (9.2%), and concordant high-risk phenotypes (2.0%). Despite normal BMI, approximately 21% of participants exhibited adiposity–metabolic discordance or combined high-risk status. The metabolic-only phenotype showed markedly adverse triglyceride, TG/HDL-c ratio, fasting glucose, and blood pressure values despite relatively preserved anthropometric parameters. Conversely, the adiposity-only phenotype exhibited increased central and visceral adiposity with comparatively preserved metabolic status. Sedentary lifestyle, unfavorable dietary adherence, smoking, older age, male sex, and higher BMI within the normal-weight range were independently associated with metabolically adverse phenotypes. The concordant high-risk phenotype demonstrated the greatest overall cardiometabolic burden. Conclusions: Adults with normal BMI are metabolically heterogeneous and may present clinically relevant hidden-risk phenotypes despite conventional normal-weight classification. Adiposity–metabolic discordance highlights the limitations of BMI-based screening alone and supports the incorporation of multidimensional anthropometric, metabolic, and lifestyle-related assessment strategies for earlier identification of cardiometabolic risk.

## 1. Introduction

Body mass index (BMI) has traditionally been considered the cornerstone for the diagnosis and classification of obesity in both clinical practice and epidemiological research. Owing to its simplicity, low cost, and reproducibility, BMI remains the most widely used anthropometric indicator for estimating adiposity-related health risk worldwide [1]. Nevertheless, an increasing body of evidence has demonstrated that BMI alone is unable to adequately reflect the complexity and heterogeneity of adipose tissue distribution, metabolic dysfunction, and cardiometabolic risk [2,3]. Individuals with similar BMI values may exhibit markedly different metabolic, inflammatory, and vascular profiles, suggesting that body weight alone provides only a partial representation of cardiometabolic health [4,5].

This limitation has become particularly evident in recent years with the recognition of metabolically unhealthy phenotypes among individuals with apparently normal body weight [6,7]. The concept of normal-weight obesity (NWO) emerged to describe individuals with normal BMI but excessive body fat accumulation, especially visceral adiposity, accompanied by metabolic alterations typically associated with obesity [8,9]. Parallel to this, the metabolically unhealthy normal-weight (MU-NW) phenotype has been increasingly associated with insulin resistance, dyslipidemia, hypertension, endothelial dysfunction, systemic inflammation, and elevated cardiovascular risk despite the absence of overt overweight or obesity according to conventional BMI thresholds [10,11,12].

The growing interest in these phenotypes reflects a broader paradigm shift in obesity research, where the focus is progressively moving from body weight toward adipose tissue quality, distribution, and functionality [13,14]. Visceral adipose tissue has been identified as a particularly pathogenic fat depot because of its endocrine, inflammatory, and lipotoxic properties [15,16]. Excess visceral fat promotes chronic low-grade inflammation, altered adipokine secretion, oxidative stress, ectopic lipid accumulation, and insulin resistance, thereby contributing to the development of cardiometabolic disease independently of total body weight [17,18]. Consequently, individuals with normal BMI but increased visceral adiposity may remain clinically underestimated while already exhibiting substantial metabolic impairment [19].

Importantly, adiposity and metabolic dysfunction do not always coexist. Some individuals may present increased visceral or dysfunctional adiposity while maintaining relatively preserved metabolic profiles, whereas others exhibit metabolic abnormalities despite limited measurable adiposity [20,21]. This phenomenon, increasingly referred to as adiposity–metabolic discordance, highlights the biological dissociation between body composition and metabolic homeostasis [22]. Such discordance challenges the traditional assumption that adiposity and cardiometabolic dysfunction necessarily progress in parallel and suggests the existence of distinct pathophysiological pathways leading to cardiometabolic disease [23].

Recent investigations have suggested that discordant phenotypes may carry different cardiovascular and metabolic prognostic implications [24,25,26]. However, despite growing interest in obesity phenotyping and precision cardiometabolic medicine, evidence regarding adiposity–metabolic discordance in normal-weight populations remains limited, particularly in large occupational cohorts [27].

In parallel, the emergence of novel adiposity-related indices has substantially improved the identification of hidden cardiometabolic risk beyond BMI [28]. Among these, the Metabolic Score for Visceral Fat (METS-VF) has shown promising ability to estimate visceral adiposity and cardiometabolic dysfunction using routinely available clinical variables [29]. Similarly, waist-related anthropometric measures such as waist-to-height ratio (WtHR) have demonstrated superior predictive performance compared with BMI for detecting cardiometabolic abnormalities and cardiovascular risk [30,31]. These markers may therefore facilitate the characterization of discordant adiposity–metabolic phenotypes among apparently healthy normal-weight individuals.

Lifestyle-related factors further contribute to this complex interaction between adiposity and metabolic health. Sedentary behavior, poor dietary quality, smoking, and reduced physical activity have all been associated with visceral fat accumulation, insulin resistance, endothelial dysfunction, and low-grade systemic inflammation independently of BMI [32,33,34,35]. Accordingly, normal-weight individuals exposed to unfavorable lifestyle patterns may develop hidden cardiometabolic alterations despite maintaining conventional weight values [36]. Understanding how these behavioral determinants interact with adiposity–metabolic discordance could provide relevant information for early risk stratification and preventive interventions.

The identification of hidden cardiometabolic risk among normal-weight adults has become increasingly relevant from a public health perspective. Current obesity definitions based primarily on BMI may fail to identify a considerable proportion of individuals at elevated cardiometabolic risk who do not meet traditional anthropometric criteria for obesity [37,38]. This limitation has recently prompted calls for a more comprehensive and clinically meaningful characterization of obesity and adiposity-related disease [39]. In this context, evaluating discordant adiposity–metabolic phenotypes may contribute to a more individualized and pathophysiologically oriented approach to cardiometabolic risk assessment.

Therefore, the present study aimed to evaluate adiposity–metabolic discordance phenotypes among adults with normal BMI in a large occupational cohort and to characterize their differential anthropometric, metabolic, vascular, and lifestyle-related profiles.

## 2. Materials and Methods

### 2.1. Study Design and Population

This cross-sectional observational study was conducted as a secondary analysis of a larger database comprising workers who underwent routine occupational health assessments between January 2019 and December 2024 in occupational health units distributed throughout Spain. Center-level identifiers, detailed geographic information, and standardized occupational-sector classifications were not available in the analytical dataset. Consequently, the number and regional distribution of participating units could not be further characterized, and potential clustering by occupational health center or occupational sector could not be specifically accounted for in the statistical analyses. For the present analysis, a subset of normal-weight participants was selected from this larger occupational health database. The source population consisted of actively employed adults aged between 18 and 69 years who underwent standardized annual medical examinations as part of mandatory occupational surveillance programs.

**Figure 1:**
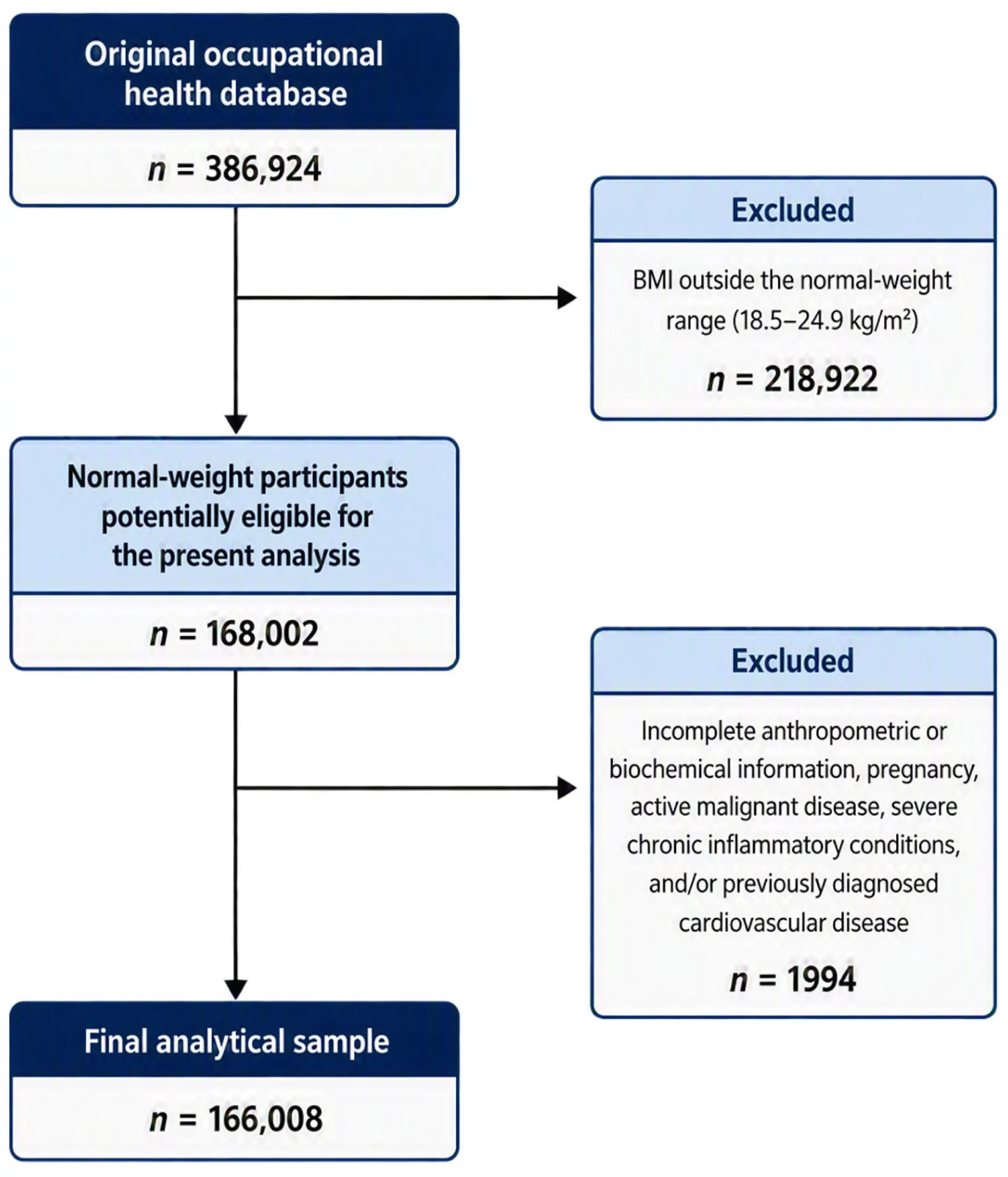
Flowchart of participant selection.

From the initial database, only participants with BMI values within the normal-weight range (18.5–24.9 kg/m^2^) were included in the present analysis. Individuals with incomplete anthropometric or biochemical information, pregnancy, active malignant disease, severe chronic inflammatory conditions, or previously diagnosed cardiovascular disease were excluded to reduce potential confounding effects on metabolic assessment. Among the 168,002 normal-weight participants potentially eligible for the present analysis, records were checked for completeness and internal consistency of the anthropometric and biochemical variables required for phenotype classification and statistical analyses. Data screening included identification of missing values and verification that recorded values were within physiologically plausible ranges. Participants were excluded if the information required for the analyses was incomplete or if they met any of the predefined exclusion criteria (pregnancy, active malignant disease, severe chronic inflammatory conditions, or previously diagnosed cardiovascular disease). Accordingly, the final analytical sample comprised complete cases for all variables required for the present analyses, and no variable-specific missing observations remained in the dataset used to generate the descriptive and inferential results. A total of 1994 participants were excluded for one or more of these reasons. Because individual participants could meet more than one exclusion criterion, exclusion counts were not disaggregated by category. The final analytical sample comprised 166,008 workers, as summarized in Figure 1.

The study followed the recommendations established for observational epidemiological research and adhered to the principles described in the Strengthening the Reporting of Observational Studies in Epidemiology (STROBE) statement [40]. Ethical procedures were conducted in accordance with the Declaration of Helsinki and current European regulations regarding biomedical research and personal data protection [41].

### 2.2. Anthropometric Assessment

Anthropometric measurements were obtained by trained occupational health professionals following standardized international procedures [42]. Body weight was measured using calibrated electronic scales with participants barefoot and wearing light clothing. Height was assessed using wall-mounted stadiometers, and BMI was subsequently calculated as weight divided by height squared (kg/m^2^).

Waist circumference was measured midway between the lower rib margin and the iliac crest at the end of a normal expiration using non-elastic anthropometric tape. Waist-to-height ratio (WtHR) was calculated by dividing waist circumference by height. According to previous evidence, WtHR ≥ 0.50 was considered indicative of increased central adiposity [43].

Blood pressure measurements were obtained in the seated position after a minimum resting period of five minutes using validated automated sphygmomanometers. Two measurements were recorded, and the mean value was used for analysis. Elevated blood pressure was defined according to current international recommendations [44].

### 2.3. Biochemical Determinations

Venous blood samples were collected after overnight fasting of at least 8–12 h. Laboratory analyses were performed in certified reference laboratories following standardized quality-control protocols [45]. Determinations included fasting plasma glucose, total cholesterol, triglycerides, high-density lipoprotein cholesterol (HDL-c), and low-density lipoprotein cholesterol (LDL-c).

Biochemical analyses were conducted using automated enzymatic methods under internal and external laboratory quality assurance systems. Because this study was based on a secondary multicenter occupational health database, laboratory-specific identifiers and longitudinal information on individual analyzers, calibration procedures, or assay changes were not available in the analytical dataset. Therefore, potential inter-laboratory or temporal variation in equipment and assay methods could not be specifically assessed beyond the standardized quality-control and laboratory quality-assurance procedures applied. The triglyceride-to-HDL cholesterol ratio (TG/HDL-c) was calculated as an indirect marker of insulin resistance and atherogenic dyslipidemia [46]. The triglyceride–glucose (TyG) index was calculated as ln [fasting triglycerides (mg/dL) × fasting glucose (mg/dL)/2] and was evaluated as a surrogate marker of insulin resistance. Direct apolipoprotein B (apoB) measurement was not available in the source occupational health database and was therefore not included in the present analyses. Nevertheless, current dyslipidemia guidelines recognize selective apoB measurement as useful for improving ASCVD risk assessment and identifying residual lipoprotein-related risk that may be underestimated by the standard lipid profile [47].

### 2.4. Definition of Adiposity–Metabolic Discordance Phenotypes

Adiposity–metabolic discordance phenotypes were established by combining adiposity-related and metabolic criteria. Increased adiposity was operationally defined as a waist-to-height ratio (WtHR) ≥ 0.50. METS-VF was additionally evaluated as a complementary marker of visceral adiposity and cardiometabolic risk [48] and was calculated using the equation originally proposed by Bello-Chavolla et al. [49]. METS-VF was not used for phenotype assignment.

Metabolic dysfunction was defined as the presence of at least two of five adverse cardiometabolic criteria: elevated triglycerides (≥150 mg/dL), reduced HDL-c (<40 mg/dL in men or <50 mg/dL in women), elevated fasting glucose (≥100 mg/dL), elevated blood pressure (systolic blood pressure ≥ 130 mmHg and/or diastolic blood pressure ≥ 85 mmHg), or an elevated TG/HDL-c ratio (≥3.0). Requiring at least two abnormal criteria was intended to avoid classifying participants as metabolically dysfunctional on the basis of a single abnormal cardiometabolic measurement. This operational approach was used to characterize metabolic health status within the adiposity–metabolic phenotype framework [50].

This operational definition was broadly aligned with previous approaches used to characterize metabolically unhealthy phenotypes, which commonly incorporate abnormalities in blood pressure, fasting glucose, triglycerides, and HDL-c and, in several studies, classify metabolic abnormality based on the presence of at least two adverse cardiometabolic components. However, no universally accepted definition of metabolic health exists, and substantial heterogeneity in the number and type of criteria has been reported across studies. Our definition additionally incorporated the TG/HDL-c ratio as a fifth criterion because of its potential value as an indicator of insulin resistance and atherogenic dyslipidemia. Accordingly, the definition used in the present study should be regarded as an operational research definition rather than a universally established diagnostic criterion [51].

Because triglycerides, HDL-c, and the TG/HDL-c ratio represent partially overlapping lipid-related information, the inclusion of these variables as separate criteria may increase the probability of meeting the definition of metabolic dysfunction.

Based on the coexistence or absence of adiposity and metabolic dysfunction, participants were classified into four phenotypes:

1. Concordant healthy phenotype (absence of adiposity and metabolic dysfunction);
2. Adiposity-only phenotype;
3. Metabolic-only phenotype;
4. Concordant high-risk phenotype (presence of both adiposity and metabolic dysfunction).

This approach allowed characterization of biological discordance between adiposity burden and metabolic status within individuals presenting normal BMI values.

### 2.5. Lifestyle-Related Variables

Lifestyle-related variables were collected using the same standardized questionnaire and assessment protocol across all participating occupational health centers. Smoking status was systematically recorded for all participants and categorized as current smoker or non-smoker. The non-smoker category included individuals who had never smoked and former smokers who had abstained from smoking for at least 12 months. Consequently, the available data did not permit separate analysis of never-smokers and former smokers or evaluation of the potential residual risk associated with recent smoking cessation.

Physical activity was assessed using the International Physical Activity Questionnaire (IPAQ), which was administered according to the same standardized protocol across all participating centers. Participants reporting a total physical activity level ≥ 600 MET-min/week were classified as physically active, whereas those with <600 MET-min/week were classified as sedentary [52]. Adherence to the Mediterranean diet was assessed using the validated 14-item Mediterranean Diet Adherence Screener (MEDAS-14), rather than a food-frequency questionnaire or food diary. The screener assesses key components of the Mediterranean dietary pattern, including the use of olive oil as the principal culinary fat; consumption of vegetables, fruits, legumes, fish or seafood, nuts, wine, sugar-sweetened beverages, commercial pastries, and sofrito; and intake of red or processed meat, butter, margarine or cream. Each item meeting the predefined Mediterranean diet criterion contributes one point, resulting in a total score ranging from 0 to 14, with higher scores indicating greater adherence to the Mediterranean dietary pattern. In the present study, a score ≥ 9 was operationally defined as favorable dietary adherence, whereas a score < 9 was classified as unfavorable dietary adherence [53,54]. MEDAS-14 is a previously validated instrument and has been extensively used for assessment of Mediterranean diet adherence in epidemiological and clinical research. Because smoking status, physical activity, and dietary adherence were self-reported, these variables were potentially subject to recall and social-desirability bias. To reduce measurement heterogeneity, the same standardized questionnaire protocol and predefined classification criteria were applied across participating occupational health centers.

### 2.6. Statistical Analysis

No a priori sample size or power calculation was performed because this study was a secondary analysis of an existing occupational health database. The final sample size was determined by the number of available records meeting the predefined eligibility criteria during the study period.

Continuous variables are presented as mean ± standard deviation, whereas categorical variables are expressed as absolute frequencies and percentages. Normality was assessed using the Kolmogorov–Smirnov test.

Comparisons between discordant phenotypes were performed using Student’s *t* test or analysis of variance (ANOVA) for continuous variables and chi-square tests for categorical variables. Effect sizes were quantified using eta squared (η^2^) for continuous variables compared across the four phenotypes by one-way ANOVA and Cramer’s V for categorical variables, in order to provide an assessment of the magnitude of between-group differences beyond statistical significance [55].

Multivariable logistic regression models were developed to evaluate the associations of age, sex, BMI, sedentary lifestyle, unfavorable dietary adherence, and smoking with discordant adiposity–metabolic phenotypes. Each variable was evaluated as an independent exposure while accounting for the other covariates included in the corresponding multivariable model. Odds ratios (ORs) and 95% confidence intervals (95% CI) were calculated. Multicollinearity among model covariates was assessed using variance inflation factors (VIFs) and tolerance values. No evidence of problematic multicollinearity was observed (VIF range: 1.02–2.27; tolerance range: 0.44–0.98). Receiver operating characteristic (ROC) curve analyses were performed to evaluate the ability of BMI, waist circumference, waist-to-height ratio (WtHR), and METS-VF to discriminate the presence of metabolic dysfunction, defined as ≥2 of the five prespecified cardiometabolic criteria. Discriminatory performance was quantified using the area under the curve (AUC) with 95% confidence intervals. Optimal cut-off values were identified using the Youden index, and the corresponding sensitivity and specificity were calculated [56].

Model adequacy was further assessed using Nagelkerke’s pseudo-R^2^, the Hosmer–Lemeshow goodness-of-fit test, and the area under the receiver operating characteristic curve (AUC). Given the large sample size and the known sensitivity of the Hosmer–Lemeshow test to minor departures from perfect calibration in large datasets, model adequacy was interpreted jointly across these measures rather than on the basis of the Hosmer–Lemeshow test alone.

A sensitivity analysis was additionally performed using an alternative definition of metabolic dysfunction that excluded the TG/HDL-c ratio, thereby reducing potential overlap among lipid-related criteria. An exploratory sex-stratified subgroup analysis was also performed to assess whether the distribution of adiposity–metabolic phenotypes and the principal multivariable associations were consistent in men and women. Within each sex stratum, logistic regression models were adjusted for age, BMI, sedentary lifestyle, unfavorable dietary adherence, and current smoking, using the concordant healthy phenotype as the reference category. Analyses were performed using complete cases, and no imputation of missing data was undertaken. No formal adjustment for multiple comparisons was applied because the analyses were primarily descriptive and exploratory. Given the large sample size, statistical significance was interpreted together with effect-size estimates and the magnitude and consistency of the observed associations. All analyses were conducted using IBM SPSS Statistics version 30.0 (IBM Corp., Armonk, NY, USA). Statistical significance was established at *p* < 0.05.

## 3. Results

### 3.1. Study Population and Distribution of Adiposity–Metabolic Phenotypes

A total of 166,008 normal-weight adults were included in the final analysis. Despite all participants having BMI values within the conventional normal range, marked heterogeneity was observed when adiposity status and metabolic dysfunction were jointly considered.

Four adiposity–metabolic phenotypes were identified. The concordant healthy phenotype was the most frequent subgroup, including 131,010 participants (78.9%). The adiposity-only phenotype included 16,282 participants (9.8%), whereas the metabolic-only phenotype included 15,354 participants (9.2%). Finally, the concordant high-risk phenotype, characterized by both increased adiposity and metabolic dysfunction, was observed in 3362 participants (2.0%).

Most participants belonged to the concordant healthy phenotype, representing nearly four-fifths of the study population. Nevertheless, approximately one in every five normal-weight adults showed some degree of adiposity–metabolic discordance or combined high-risk status. Isolated adiposity and isolated metabolic dysfunction were observed with similar frequencies, highlighting that excess visceral adiposity and metabolic impairment do not necessarily coexist in individuals with normal BMI values. Although the concordant high-risk phenotype was the least prevalent subgroup, it identified a clinically relevant subset of participants simultaneously presenting increased adiposity and metabolic dysfunction despite apparently normal body weight.

In a sensitivity analysis excluding the TG/HDL-c ratio from the metabolic dysfunction criteria, 12,736 participants (7.7%) met the alternative definition of metabolic dysfunction, compared with 18,716 (11.3%) under the primary five-component definition. Under this alternative definition, the prevalence of the concordant healthy, adiposity-only, metabolic-only, and concordant high-risk phenotypes was 81.9%, 10.5%, 6.3%, and 1.4%, respectively. Thus, exclusion of the TG/HDL-c ratio reduced the estimated prevalence of metabolically adverse phenotypes, while adiposity–metabolic discordance remained evident. In exploratory sex-stratified analyses, adiposity–metabolic discordance remained evident in both men and women, although phenotype distribution differed by sex (χ^2^ = 9242.77, *p* < 0.001; Cramer’s V = 0.236). Among men, 69.6% were classified as concordant healthy, 15.3% as adiposity-only, 11.7% as metabolic-only, and 3.4% as concordant high-risk; the corresponding proportions among women were 88.0%, 4.5%, 6.9%, and 0.7%, respectively. In sex-stratified multivariable models, sedentary lifestyle remained strongly associated with metabolically adverse phenotypes in both sexes. For the metabolic-only phenotype, the adjusted OR was 28.74 (95% CI 26.07–31.69) in men and 12.03 (95% CI 10.92–13.25) in women; for the concordant high-risk phenotype, the corresponding ORs were 30.20 (95% CI 25.48–35.81) and 15.61 (95% CI 10.94–22.27), respectively. These findings indicate that the principal pattern of association was preserved in both sexes, although the magnitude of some associations differed (Supplementary Table S1).

**Figure 2:**
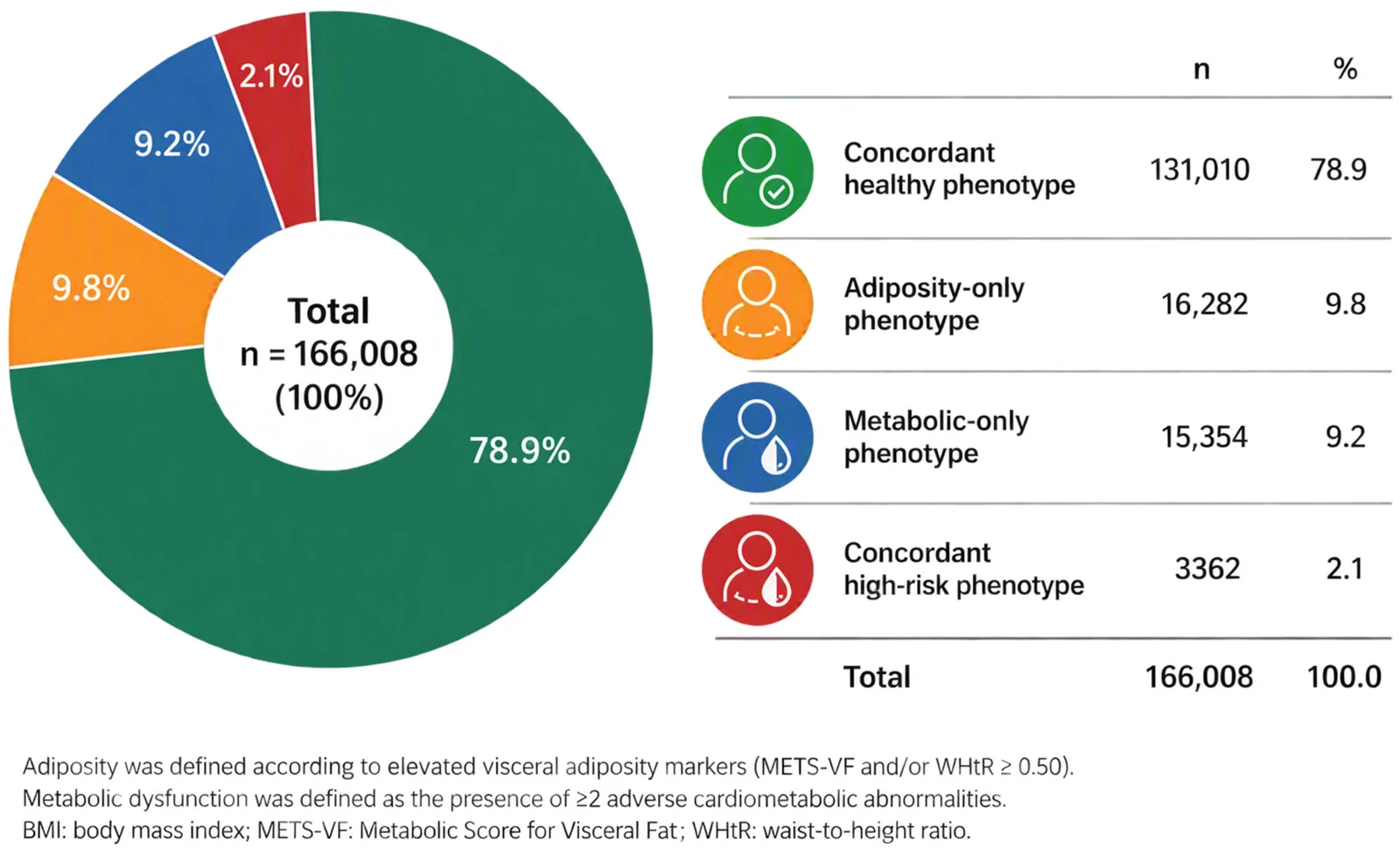
Distribution of adiposity–metabolic discordance phenotypes in normal-weight adults.

The proportional distribution of adiposity–metabolic discordance phenotypes is illustrated in Figure 2.

Overall, 11.8% of participants showed increased adiposity, whereas 11.3% fulfilled criteria for metabolic dysfunction. Importantly, these two dimensions did not completely overlap, since a substantial proportion of individuals exhibited either isolated adiposity or isolated metabolic dysfunction. This confirms the existence of adiposity–metabolic discordance within the normal BMI range.

### 3.2. Demographic and Anthropometric Characteristics Across Phenotypes

Demographic and anthropometric characteristics differed markedly across adiposity–metabolic phenotypes. Participants in the concordant healthy group were younger than those in the metabolic-only and concordant high-risk phenotypes. Mean age increased from 36.17 ± 9.45 years in the concordant healthy phenotype to 44.13 ± 10.35 years in the concordant high-risk phenotype.

BMI values remained within the normal range in all groups but showed a progressive increase across phenotypes. Mean BMI was 22.28 ± 1.69 kg/m^2^ in the concordant healthy group, 23.16 ± 1.34 kg/m^2^ in the adiposity-only group, 22.91 ± 1.50 kg/m^2^ in the metabolic-only group, and 23.51 ± 1.17 kg/m^2^ in the concordant high-risk group.

Waist circumference and waist-to-height ratio showed the clearest separation between adiposity-defined phenotypes. Waist circumference increased from 74.07 ± 6.76 cm in the concordant healthy group to 86.96 ± 6.09 cm in the concordant high-risk group. Similarly, WtHR increased from 0.44 ± 0.03 to 0.52 ± 0.03 across these phenotypes.

Demographic, anthropometric, glycemic, lipid, and vascular characteristics according to adiposity–metabolic phenotype are shown in Table 1.

Marked anthropometric differences were observed across adiposity–metabolic phenotypes. As expected from the WtHR-based definition of increased adiposity, adiposity-related phenotypes showed higher WtHR values. Beyond this classification-related difference, the concordant high-risk phenotype exhibited the highest BMI, METS-VF, and blood pressure values. Age and male predominance progressively increased from the concordant healthy phenotype toward metabolically adverse phenotypes, suggesting an unfavorable demographic gradient associated with adiposity–metabolic discordance.

### 3.3. Metabolic and Vascular Profile According to Adiposity–Metabolic Phenotype

Clear differences in metabolic and vascular parameters were observed across phenotypes. The concordant healthy phenotype showed the most favorable profile, whereas the concordant high-risk phenotype showed the most adverse cardiometabolic pattern.

Fasting glucose increased from 82.61 ± 10.60 mg/dL in the concordant healthy phenotype to 91.60 ± 13.96 mg/dL in the concordant high-risk phenotype. Triglyceride concentrations showed an even more pronounced gradient, rising from 76.81 ± 28.15 mg/dL in the concordant healthy group to 182.70 ± 96.51 mg/dL in the concordant high-risk group. HDL cholesterol showed the opposite pattern, decreasing from 54.87 ± 6.89 mg/dL to 47.68 ± 7.11 mg/dL. Consistent with these findings, the TyG index increased progressively across phenotypes, from 7.99 ± 0.40 in the concordant healthy phenotype to 8.89 ± 0.51 in the concordant high-risk phenotype (*p* < 0.001; η^2^ = 0.277).

Blood pressure values also differed substantially. Systolic blood pressure increased from 113.46 ± 12.82 mmHg in the concordant healthy phenotype to 127.43 ± 16.15 mmHg in the concordant high-risk phenotype. Diastolic blood pressure increased from 68.10 ± 9.10 mmHg to 76.95 ± 10.40 mmHg.

The metabolic and vascular profile across phenotypes is summarized in Table 2.

Substantial metabolic and vascular differences were identified across adiposity–metabolic phenotypes. The concordant healthy phenotype consistently showed the most favorable cardiometabolic profile, whereas the concordant high-risk phenotype exhibited the highest fasting glucose, triglycerides, TG/HDL-c ratio, TyG index, blood pressure, and METS-VF values together with the lowest HDL cholesterol concentrations. Importantly, the metabolic-only phenotype demonstrated markedly adverse lipid and glycemic parameters despite relatively preserved adiposity-related measures, suggesting that clinically relevant metabolic dysfunction may develop independently of overt visceral adiposity. The largest effect sizes across phenotypes were observed for the TG/HDL-c ratio, METS-VF, and triglycerides, while the TyG index also showed a substantial between-phenotype effect size (η^2^ = 0.277), further supporting marked differences in metabolic risk across phenotypes.

**Figure 3:**
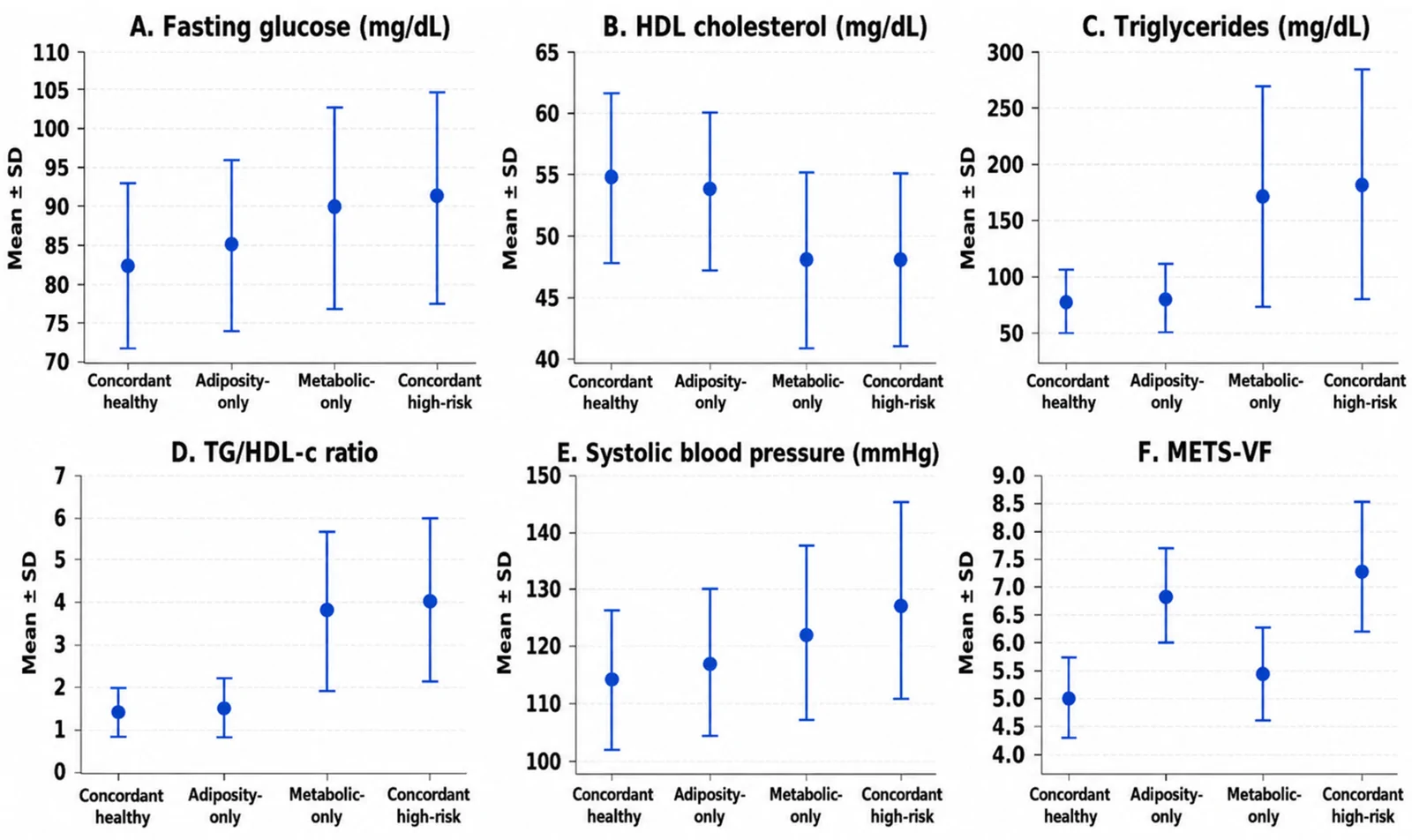
Cardiometabolic profile across adiposity–metabolic phenotypes. Mean ± standard deviation values of (**A**) fasting glucose, (**B**) HDL cholesterol, (**C**) triglycerides, (**D**) TG/HDL-c ratio, (**E**) systolic blood pressure, and (**F**) METS-VF are shown for the concordant healthy, adiposity-only, metabolic-only, and concordant high-risk phenotypes. Each panel displays values in the original measurement scale of the corresponding variable. TG/HDL-c: triglyceride-to-HDL cholesterol ratio; METS-VF: Metabolic Score for Visceral Fat.

Differences in metabolic and vascular parameters across adiposity–metabolic phenotypes are illustrated in Figure 3.

### 3.4. Clinical Relevance of Discordant Phenotypes

**Figure 4:**
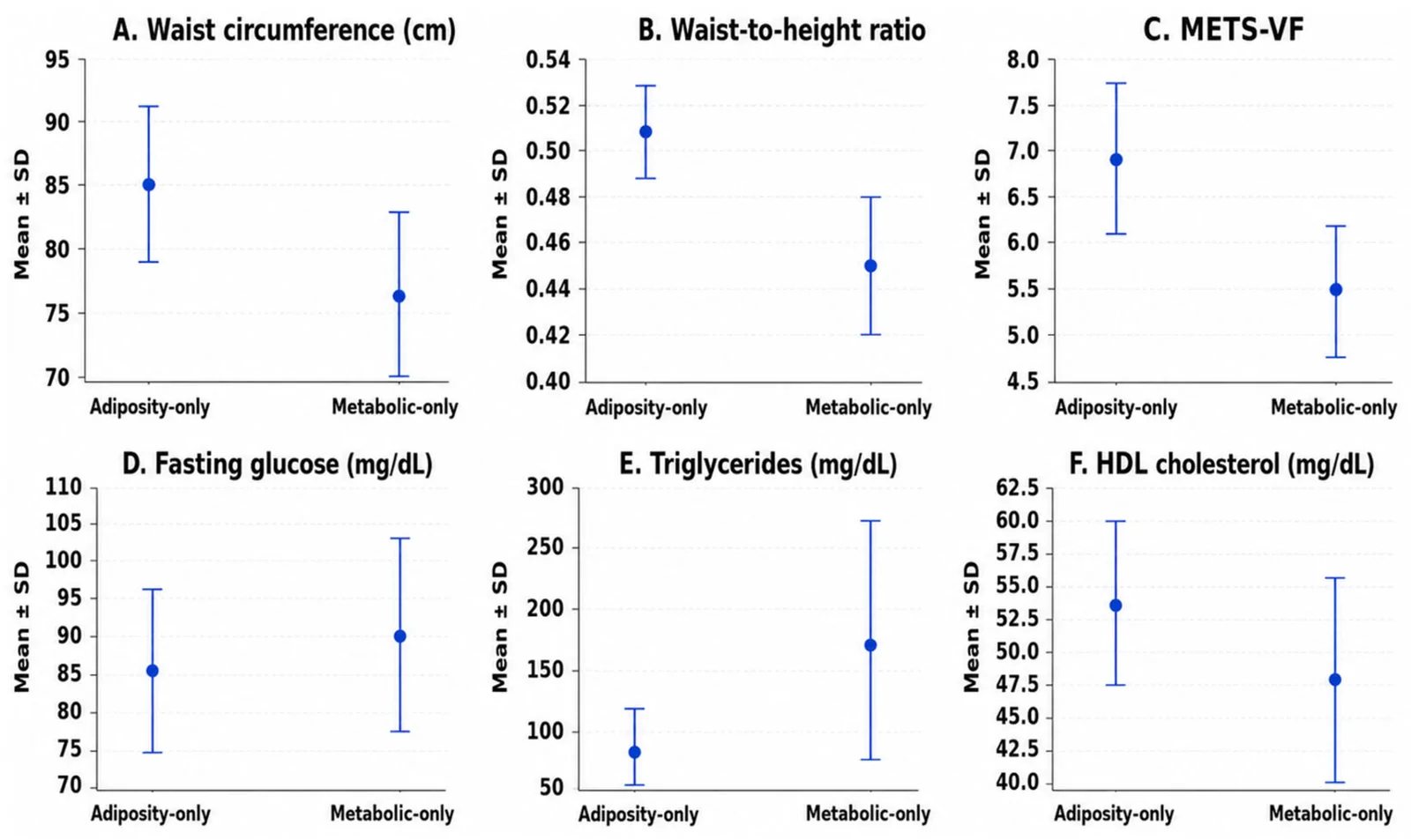
Comparative anthropometric and metabolic profiles of the two discordant adiposity–metabolic phenotypes. Mean ± standard deviation values of (**A**) waist circumference, (**B**) waist-to-height ratio (WtHR), (**C**) METS-VF, (**D**) fasting glucose, (**E**) triglycerides, and (**F**) HDL cholesterol are shown for the adiposity-only and metabolic-only phenotypes. Each panel displays values in the original measurement scale of the corresponding variable. WtHR: waist-to-height ratio; METS-VF: Metabolic Score for Visceral Fat; HDL: high-density lipoprotein.

The two discordant phenotypes showed clearly different clinical patterns (Figure 4). The adiposity-only phenotype was characterized by higher BMI, waist circumference, WtHR, and METS-VF values but relatively preserved metabolic parameters, whereas the metabolic-only phenotype showed normal WtHR values but markedly worse triglycerides, HDL cholesterol, fasting glucose, and blood pressure. In particular, triglyceride concentrations were substantially higher in the metabolic-only than in the adiposity-only phenotype (171.28 ± 95.88 vs. 81.17 ± 29.50 mg/dL), while HDL cholesterol concentrations were lower (47.79 ± 7.51 vs. 53.80 ± 6.34 mg/dL) (Figure 4). These contrasting anthropometric and metabolic profiles illustrate the distinct nature of the two discordant phenotypes and indicate that metabolic dysfunction may be present despite the absence of increased central adiposity.

### 3.5. Lifestyle-Related Characteristics Across Phenotypes

Lifestyle-related factors differed markedly across adiposity–metabolic phenotypes. The concordant healthy group showed the highest prevalence of favorable behaviors, whereas metabolic-only and concordant high-risk phenotypes showed the least favorable lifestyle profile.

Being physically active was reported by 86.4% of participants in the concordant healthy phenotype, compared with 81.6% in the adiposity-only phenotype, 19.0% in the metabolic-only phenotype, and 15.9% in the concordant high-risk phenotype.

Favorable dietary adherence showed a similar pattern, decreasing from 79.4% in the concordant healthy phenotype to 18.1% in the concordant high-risk phenotype. Current smoking was more frequent among metabolically adverse phenotypes, affecting 50.4% of participants in the metabolic-only group and 52.9% in the concordant high-risk group.

Lifestyle-related characteristics across adiposity–metabolic phenotypes are summarized in Table 3.

**Figure 5:**
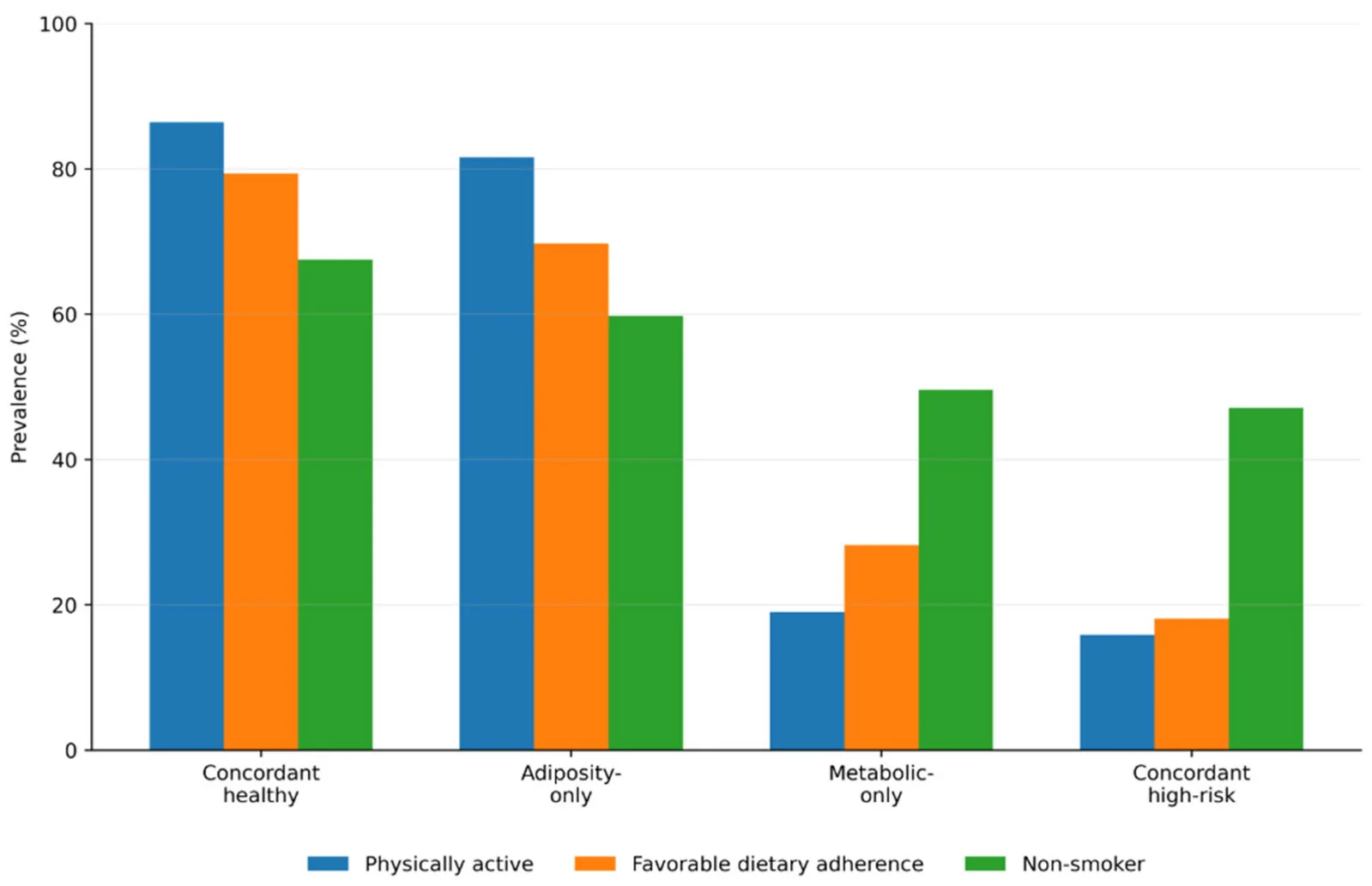
Lifestyle-related characteristics according to adiposity–metabolic discordance phenotypes.

Lifestyle-related behaviors across adiposity–metabolic discordance phenotypes are illustrated in Figure 5.

### 3.6. Prevalence of Adverse Cardiometabolic Outcomes

The prevalence of adverse cardiometabolic outcomes increased markedly across phenotypes. Hypertension was present in 5.1% of the concordant healthy phenotype, 7.6% of the adiposity-only phenotype, 22.6% of the metabolic-only phenotype, and 26.2% of the concordant high-risk phenotype.

Impaired fasting glucose was observed in 4.2% of concordant healthy participants, 6.5% of adiposity-only participants, 29.6% of metabolic-only participants, and 31.4% of concordant high-risk participants.

Elevated TG/HDL ratio was almost absent in concordant healthy and adiposity-only phenotypes but highly prevalent in metabolically adverse groups, affecting 67.2% of the metabolic-only phenotype and 71.7% of the concordant high-risk phenotype.

The prevalence of adverse cardiometabolic outcomes across adiposity–metabolic phenotypes is presented in Table 4.

### 3.7. Discriminatory Performance of Anthropometric and Metabolic Indices

ROC curve analyses were performed to assess the ability of selected anthropometric and metabolic indices to identify participants with metabolic dysfunction. METS-VF showed the highest discriminatory performance among the evaluated indices and provided the most balanced sensitivity and specificity (Table 5). However, the higher discriminatory performance observed for METS-VF should be interpreted cautiously because its calculation incorporates metabolic variables that are related to components of the metabolic dysfunction definition. Therefore, this finding reflects discrimination within the present operational framework and should not be interpreted as evidence of independent diagnostic superiority.

### 3.8. Multivariable Analysis of Adiposity–Metabolic Phenotypes

Multivariable logistic regression analyses showed that age, male sex, BMI, sedentary behavior, poor dietary adherence, and current smoking were associated with discordant and high-risk phenotypes, although the magnitude of association differed substantially across groups.

Compared with the concordant healthy phenotype, male sex and higher BMI were independently associated with all three discordant or high-risk phenotypes. Sedentary lifestyle showed by far the strongest association with the metabolically adverse phenotypes, with adjusted ORs of 20.40 (95% CI 19.04–21.85) for the metabolic-only phenotype and 26.11 (95% CI 22.49–30.32) for the concordant high-risk phenotype. Crude estimates were even larger (OR 27.09 and 33.54, respectively), indicating that multivariable adjustment attenuated but did not eliminate these strong associations.

Current smoking was not independently associated with the adiposity-only phenotype but remained associated with the metabolic-only and concordant high-risk phenotypes. Unfavorable dietary adherence showed strong crude associations with the metabolically adverse phenotypes; however, these associations were markedly attenuated after simultaneous adjustment for the other demographic, anthropometric, and lifestyle-related factors.

Crude and adjusted associations between demographic, anthropometric, and lifestyle-related variables and adiposity–metabolic discordance phenotypes are shown in Table 6.

Model adequacy differed across phenotype comparisons. Nagelkerke’s R^2^ values were 0.126, 0.430, and 0.427 for the adiposity-only, metabolic-only, and concordant high-risk models, respectively. Discriminatory performance was acceptable for the adiposity-only model (AUC 0.727; 95% CI 0.723–0.732), good for the metabolic-only model (AUC 0.885; 95% CI 0.882–0.889), and excellent for the concordant high-risk model (AUC 0.938; 95% CI 0.932–0.944). The Hosmer–Lemeshow test was statistically significant for all three models (all *p* < 0.001); given the very large sample size, these results were interpreted together with the pseudo-R^2^ and discrimination measures rather than as an isolated indicator of model inadequacy.

**Figure 6:**
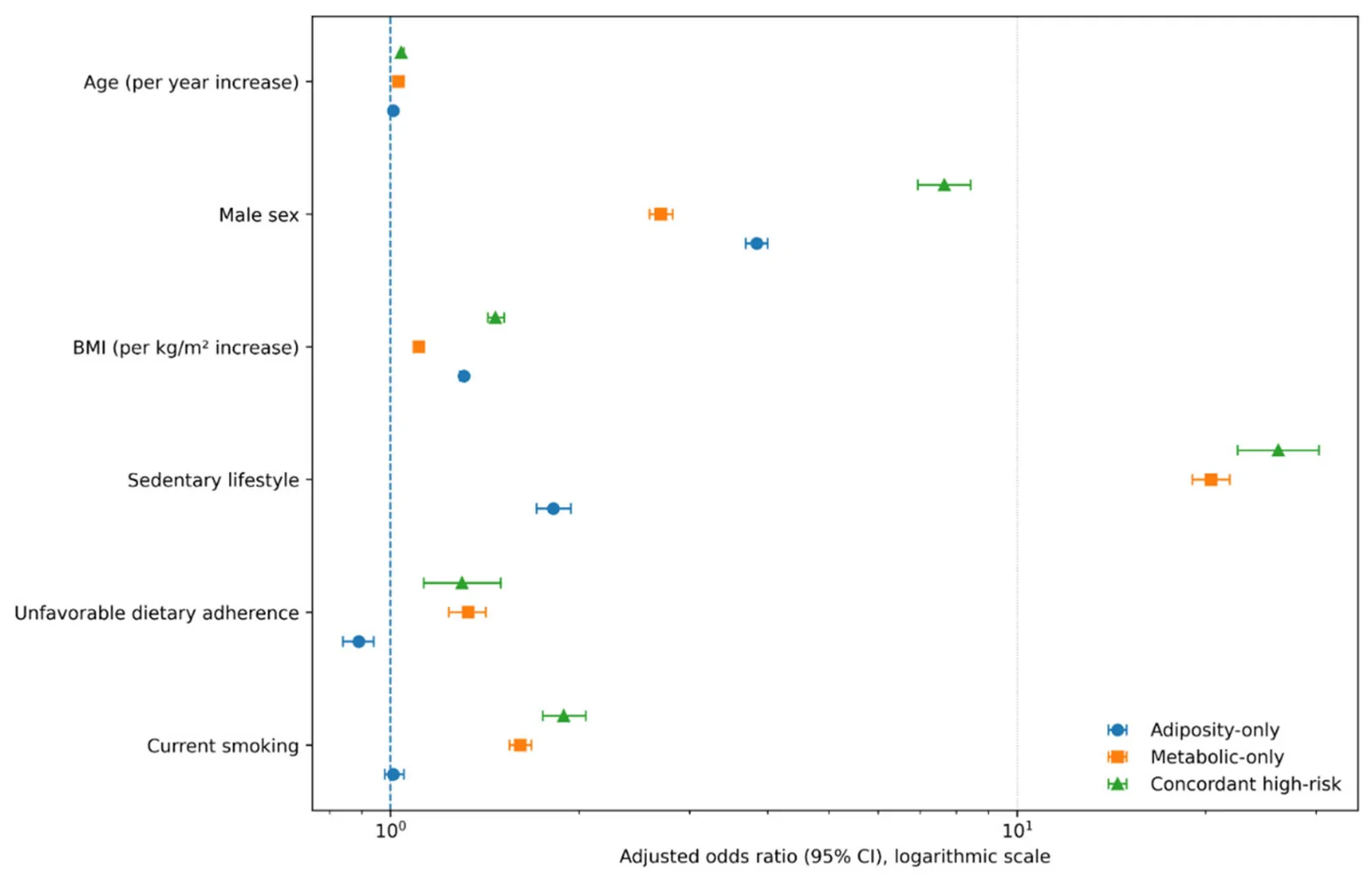
Adjusted odds ratios (ORs) and 95% confidence intervals (95% CI) for adiposity–metabolic discordance phenotypes according to demographic, anthropometric, and lifestyle-related factors. Adjusted ORs were derived from multivariable logistic regression models simultaneously including age, sex, body mass index (BMI), sedentary lifestyle, unfavorable dietary adherence, and current smoking, with the concordant healthy phenotype as the reference category. The vertical dashed line indicates the null value (OR = 1). The *x*-axis is displayed on a logarithmic scale.

The adjusted associations between demographic, anthropometric, and lifestyle-related factors and adiposity–metabolic discordance phenotypes are illustrated in Figure 6.

### 3.9. Integrated Summary of Findings

Overall, the results demonstrate that adults with normal BMI are not metabolically homogeneous. A substantial proportion exhibited discordance between adiposity and metabolic status, with either isolated adiposity or isolated metabolic dysfunction.

The metabolic-only phenotype was particularly relevant because it identified individuals without central adiposity who nevertheless showed marked dyslipidemia, higher glucose levels, higher blood pressure, and unfavorable lifestyle patterns. Conversely, the adiposity-only phenotype showed increased central adiposity but relatively preserved metabolic status, suggesting that adiposity burden and metabolic impairment may follow partially independent pathways.

The concordant high-risk phenotype showed the most adverse overall profile, combining increased adiposity, metabolic dysfunction, older age, male predominance, unfavorable lifestyle behaviors, and the highest prevalence of hypertension, impaired fasting glucose, and elevated TG/HDL ratio.

These findings support the existence of biologically meaningful adiposity–metabolic discordance among normal-weight adults and suggest that BMI-based classification alone may fail to identify clinically relevant hidden-risk profiles.

## 4. Discussion

### 4.1. Principal Findings

The present study provides further evidence that substantial cardiometabolic heterogeneity exists among adults classified as normal weight according to conventional BMI criteria. In a large occupational cohort of more than 166,000 Spanish workers, we identified four distinct adiposity–metabolic phenotypes, including two biologically discordant groups characterized by either isolated adiposity accumulation or isolated metabolic dysfunction despite BMI values within the normal range. Importantly, both discordant phenotypes exhibited clinically relevant adverse profiles, although with markedly different anthropometric, metabolic, and behavioral characteristics.

Several findings deserve particular attention. First, approximately one in every five normal-weight participants showed some degree of adiposity–metabolic discordance or combined high-risk phenotype, indicating that normal BMI does not necessarily reflect preserved cardiometabolic health. Second, the metabolic-only phenotype exhibited markedly adverse glycemic, lipid, and vascular parameters despite relatively preserved anthropometric indices, reinforcing the concept that metabolic dysfunction may develop independently of overt visceral adiposity. Third, unfavorable lifestyle-related behaviors, particularly sedentary lifestyle and poor dietary adherence, were strongly associated with metabolically adverse phenotypes. Finally, the concordant high-risk phenotype demonstrated the most severe cardiometabolic burden, suggesting a cumulative detrimental interaction between visceral adiposity, metabolic impairment, and adverse lifestyle exposures.

Taken together, these observations support the growing shift from weight-centered approaches toward multidimensional cardiometabolic phenotyping and reinforce the limitations of relying exclusively on BMI for cardiometabolic risk stratification [57].

### 4.2. Adiposity–Metabolic Discordance and Hidden Cardiometabolic Risk

One of the most relevant observations of the present study is the clear biological dissociation identified between adiposity burden and metabolic dysfunction within the normal-weight range. Traditionally, excess adiposity and metabolic impairment have been considered parallel manifestations of the same pathological process. However, emerging evidence suggests that adipose tissue dysfunction, ectopic fat deposition, inflammation, insulin resistance, and vascular impairment may evolve through partially independent mechanisms [58,59].

Our findings align with recent investigations demonstrating that metabolically adverse phenotypes may occur in the absence of overt obesity and may remain clinically underestimated when BMI is used as the principal screening tool [60,61]. In particular, the metabolic-only phenotype identified in our cohort exhibited substantially elevated triglycerides, TG/HDL-c ratio, fasting glucose, and blood pressure despite comparatively preserved waist-related anthropometric markers. This observation may reflect early adipose tissue dysfunction, altered lipid partitioning, reduced insulin sensitivity, hepatic steatosis, or low-grade inflammatory activation not fully captured by conventional anthropometric measurements [62,63]. The marked glycemic and lipid abnormalities observed in the metabolic-only phenotype further emphasize the close interplay between glucose and lipid metabolism in determining cardiometabolic risk. Previous evidence has highlighted the interdependence of these metabolic pathways and shown that they may respond differently to specific nutritional exposures, reinforcing the importance of considering glycemic and lipid disturbances jointly rather than as isolated abnormalities [64]. In this context, our findings suggest that metabolic abnormalities may identify a clinically relevant risk phenotype even in normal-weight individuals who would otherwise be considered at relatively low risk on the basis of anthropometric criteria alone.

Conversely, participants classified within the adiposity-only phenotype presented increased visceral adiposity markers but comparatively preserved metabolic parameters. This phenotype may represent an intermediate or transitional stage preceding overt metabolic deterioration, although longitudinal studies are still needed to clarify its long-term cardiovascular prognosis [65]. Previous evidence has suggested that some individuals may temporarily maintain preserved metabolic homeostasis despite increased adiposity because of greater adipose tissue expandability, lower ectopic lipid deposition, preserved mitochondrial function, or reduced inflammatory activation [66,67]. Nevertheless, accumulating data indicate that these apparently “metabolically preserved” phenotypes may progressively lose their protective profile over time [68].

The magnitude of the between-phenotype differences should also be considered from a clinical rather than solely statistical perspective, particularly given the large sample size. Differences in triglycerides, HDL cholesterol, fasting glucose, and blood pressure were accompanied by substantial differences in the prevalence of clinically adverse states, including hypertension, impaired fasting glucose, and an elevated TG/HDL-c ratio. For example, hypertension increased from 5.1% in the concordant healthy phenotype to 22.6% and 26.2% in the metabolic-only and concordant high-risk phenotypes, respectively, while impaired fasting glucose increased from 4.2% to 29.6% and 31.4%. Similarly, an elevated TG/HDL-c ratio was present in 67.2% and 71.7% of the metabolically adverse phenotypes compared with 3.1% of the concordant healthy phenotype. These differences suggest clinically meaningful cardiometabolic separation between phenotypes rather than merely statistically significant variation. In contrast, smaller between-group differences in individual continuous biomarkers should be interpreted more cautiously, as statistical significance in this large cohort does not necessarily imply clinical importance for each measure considered in isolation.

Collectively, these findings indicate that cardiometabolic health cannot be inferred from BMI-defined weight status alone, as individuals within the normal BMI range may exhibit substantial heterogeneity in adiposity distribution and metabolic risk [69].

### 4.3. Visceral Adiposity, METS-VF, and Metabolic Dysfunction

The present study additionally highlights the relevance of visceral adiposity-related markers for identifying hidden cardiometabolic risk among adults with normal BMI. Waist-to-height ratio and METS-VF showed substantial differences across adiposity–metabolic phenotypes and were associated with adverse metabolic profiles.

Visceral adipose tissue is increasingly recognized as one of the principal drivers of cardiometabolic dysfunction because of its endocrine and inflammatory activity [70]. Unlike subcutaneous adipose tissue, visceral fat exhibits greater lipolytic activity and promotes increased free fatty acid flux toward the liver, contributing to hepatic insulin resistance, dyslipidemia, endothelial dysfunction, oxidative stress, and chronic low-grade inflammation [71,72]. Consequently, individuals with apparently normal body weight but increased visceral adiposity may already present clinically relevant metabolic alterations despite remaining below traditional BMI thresholds.

These findings can also be interpreted within the emerging cardiovascular–kidney–metabolic (CKM) framework, which conceptualizes metabolic dysfunction, cardiovascular disease, and kidney disease as interconnected components of a progressive multisystem continuum. Within this framework, adiposity and metabolic abnormalities such as insulin resistance, dyslipidemia, hypertension, and impaired glucose regulation contribute to a broader pathophysiological network linking metabolic dysfunction with subsequent cardiovascular and kidney disease [73]. From this perspective, the adiposity–metabolic discordance identified in our normal-weight population may represent an early manifestation of cardiometabolic vulnerability that would not necessarily be recognized by BMI-based screening alone. This interpretation further supports the potential value of integrating anthropometric and metabolic information for earlier identification of individuals with an unfavorable cardiometabolic profile.

METS-VF has recently emerged as a practical surrogate marker for visceral adiposity and adipose tissue dysfunction integrating anthropometric and metabolic information [74]. Several recent studies have demonstrated strong associations between elevated METS-VF values and type 2 diabetes, metabolic syndrome, chronic kidney disease, non-alcoholic fatty liver disease, and cardiovascular events [75,76,77]. Our findings complement these observations by showing that variation in METS-VF is also detectable among adults within the normal BMI range and is associated with distinct adiposity–metabolic phenotypes.

In the ROC analysis, METS-VF showed the highest AUC among the indices evaluated for identifying metabolic dysfunction. However, this finding should be interpreted cautiously because METS-VF integrates metabolic information related to components used to define the outcome, resulting in partial predictor–outcome overlap. Accordingly, its higher AUC should be regarded as reflecting discriminatory performance within the present operational framework rather than as evidence of superior independent diagnostic performance.

Similarly, waist-to-height ratio showed clear progressive increases across adiposity-related phenotypes. Current evidence increasingly supports WtHR as a more sensitive marker of cardiometabolic risk than BMI because it more accurately reflects central fat distribution and visceral adiposity burden [78,79]. The present results reinforce the potential utility of combining BMI with waist-related indices and metabolic markers for more comprehensive cardiometabolic risk stratification.

### 4.4. Lifestyle-Related Determinants of Metabolic Dysfunction

Another major finding of the present study is the strong association observed between unfavorable lifestyle-related behaviors and metabolically adverse phenotypes. Sedentary lifestyle emerged as the variable most strongly associated with the metabolic-only and concordant high-risk phenotypes, with notably large odds ratios. However, the magnitude of these associations should be interpreted cautiously. Physical activity was self-reported using the IPAQ and subsequently dichotomized according to the predefined threshold (≥600 MET-min/week), which necessarily reduces the information contained in the original continuous measure. Self-reported physical activity is also susceptible to recall and reporting bias [53], and misclassification introduced by dichotomization may have contributed to the magnitude of the observed associations. Accordingly, these odds ratios should be interpreted as associations within the present operational classification rather than as precise estimates of the causal effect of physical inactivity.

Physical inactivity has consistently been associated with impaired insulin sensitivity, endothelial dysfunction, altered skeletal muscle glucose utilization, mitochondrial dysfunction, and visceral fat accumulation independently of body weight [80,81]. Recent evidence additionally suggests that prolonged sedentary behavior may directly contribute to chronic inflammation, impaired lipid metabolism, and vascular dysfunction even among individuals with normal BMI values [82]. In our cohort, sedentary participants showed particularly adverse triglyceride and HDL cholesterol profiles, supporting the close relationship between physical inactivity and early atherogenic dyslipidemia.

Dietary adherence also demonstrated substantial associations with cardiometabolic phenotypes. Participants with unfavorable dietary patterns showed markedly higher prevalence of metabolic dysfunction, elevated TG/HDL-c ratio, and impaired fasting glucose. These observations are consistent with recent evidence supporting the protective cardiometabolic effects of Mediterranean dietary patterns rich in fruits, vegetables, legumes, fish, olive oil, and minimally processed foods [83,84]. Mediterranean diet adherence has been associated with lower visceral adiposity, improved endothelial function, reduced systemic inflammation, and lower incidence of cardiometabolic disease independently of BMI [85].

Smoking was additionally associated with adverse metabolic profiles despite comparatively smaller effect sizes. Tobacco exposure has been linked to endothelial dysfunction, oxidative stress, altered lipid metabolism, insulin resistance, and preferential central fat accumulation despite lower total body weight [86,87]. The present findings support the concept that smoking contributes to hidden cardiometabolic impairment even among individuals without overt obesity.

Importantly, the accumulation of favorable lifestyle behaviors demonstrated a clear inverse dose–response relationship with adverse cardiometabolic outcomes. Participants exhibiting regular physical activity, favorable dietary adherence, and non-smoking status simultaneously showed substantially lower prevalence of metabolically adverse phenotypes. These findings reinforce the cumulative and synergistic protective effect of healthy lifestyle patterns on cardiometabolic health [88].

### 4.5. Clinical and Public Health Implications

The present findings may have important implications for both clinical practice and public health strategies. Current obesity screening approaches remain heavily dependent on BMI despite its well-recognized limitations for detecting visceral adiposity and metabolic dysfunction [89]. As demonstrated in our cohort, a substantial proportion of apparently normal-weight individuals may already exhibit clinically relevant metabolic impairment and increased cardiometabolic vulnerability.

Failure to identify these hidden-risk phenotypes may delay preventive interventions until overt obesity, diabetes, hypertension, or cardiovascular disease become clinically established [90]. Our results therefore support the incorporation of additional anthropometric and metabolic markers, including waist-to-height ratio, TG/HDL-c ratio, and METS-VF, into routine cardiometabolic assessment even among adults with normal BMI values.

From a preventive perspective, the strong associations observed between lifestyle-related behaviors and metabolically adverse phenotypes emphasize the relevance of early behavioral interventions independently of body weight classification. Physical activity promotion, smoking cessation, and improvement in dietary quality may provide substantial cardiometabolic benefits even in individuals who do not meet traditional overweight or obesity criteria [91].

The occupational setting may represent a particularly valuable environment for implementing early multidimensional cardiometabolic screening strategies because large populations of apparently healthy adults can be evaluated before the development of overt disease [92].

### 4.6. Strengths and Limitations

The present study has several strengths. First, the exceptionally large sample size provided substantial statistical power and allowed detailed characterization of multiple adiposity–metabolic phenotypes within the normal-weight range. Second, the study incorporated comprehensive anthropometric, metabolic, vascular, and lifestyle-related information obtained through standardized occupational health evaluations. Third, the use of adiposity-related indices such as METS-VF and waist-to-height ratio allowed a more refined assessment of hidden visceral adiposity beyond conventional BMI classification.

Nevertheless, several limitations should also be acknowledged. The cross-sectional design prevents establishment of causal or temporal relationships between lifestyle-related factors and adiposity–metabolic phenotypes; consequently, the strong associations observed for sedentary lifestyle should not be interpreted as evidence that physical inactivity caused the metabolically adverse phenotypes. The particularly large odds ratios observed for sedentary lifestyle warrant cautious interpretation. Examination of the underlying data showed no sparse or empty exposure–outcome cells, all logistic regression models converged normally, and multicollinearity diagnostics were within acceptable ranges, providing no evidence of sparse-data bias or quasi-separation. Nevertheless, physical activity was represented as a binary category derived from self-reported questionnaire data, and residual misclassification and unmeasured confounding may have contributed to the magnitude of these associations. The operational definition of metabolic dysfunction included triglycerides, HDL-c, and the TG/HDL-c ratio as separate criteria. Because the TG/HDL-c ratio is mathematically derived from two variables that were also independently included in the definition, this approach introduces partial redundancy and may increase the estimated prevalence of metabolic dysfunction. Consistent with this possibility, the sensitivity analysis excluding the TG/HDL-c ratio resulted in lower prevalences of the metabolic-only and concordant high-risk phenotypes. Nevertheless, adiposity–metabolic discordance remained evident under the alternative definition, supporting the robustness of the main finding while indicating that absolute phenotype prevalence estimates should be interpreted in light of the operational definition used. Additionally, the ROC results for METS-VF should be interpreted cautiously because this index incorporates metabolic information related to components used to define metabolic dysfunction, resulting in partial predictor–outcome overlap. Consequently, its higher AUC should not be interpreted as evidence of independent diagnostic superiority over the other anthropometric indices evaluated. Lifestyle-related variables were obtained through self-reported questionnaires and may therefore be affected by recall bias or misclassification. The occupational nature of the cohort may also introduce a healthy worker effect, as actively employed individuals are generally healthier than the general population. This selection mechanism could lead to underestimation of the absolute prevalence of adverse metabolic phenotypes and limits extrapolation of the observed prevalence estimates to the general adult population. In addition, no formal adjustment for multiple comparisons was applied because the analyses were primarily descriptive and exploratory. Given the large sample size and number of statistical comparisons, some statistically significant findings, particularly those with small effect sizes, may reflect chance or have limited clinical relevance. Accordingly, statistical significance was interpreted together with effect-size estimates and the magnitude and consistency of the observed associations. Additionally, direct imaging techniques for visceral adiposity assessment, such as magnetic resonance imaging or computed tomography, were not available in the present occupational setting. Direct apoB measurement was not available in the source database and could therefore not be evaluated. This represents an additional limitation, as current dyslipidemia guidelines recognize selective apoB measurement as useful for refining ASCVD risk assessment and identifying residual lipoprotein-related risk that may be underestimated by the standard lipid profile. Furthermore, the lack of center-level, detailed geographic, standardized occupational-sector, and laboratory-specific identifiers precluded assessment of potential clustering effects and limited a more detailed evaluation of geographic and occupational representativeness and inter-laboratory variability. Longitudinal information on specific analyzers, calibration procedures, or assay changes across the study period was also unavailable. Finally, because the study population consisted predominantly of actively employed adults, a healthy worker effect cannot be excluded. Working populations may be healthier than the general population because individuals with severe illness or functional limitations are less likely to remain actively employed. Consequently, the prevalence of adverse cardiometabolic phenotypes may be underestimated, and the findings may not be fully generalizable to elderly, institutionalized, severely ill, or non-working populations.

## 5. Conclusions

Adiposity–metabolic discordance is common among adults with normal BMI and identifies biologically distinct phenotypes with markedly different cardiometabolic profiles. Metabolic dysfunction may occur independently of overt visceral adiposity, whereas isolated adiposity accumulation may coexist with relatively preserved metabolic status. Unfavorable lifestyle-related behaviors, particularly sedentary lifestyle, are strongly associated with metabolically adverse phenotypes despite apparently normal body weight.

These findings reinforce the limitations of BMI-based screening alone and support the incorporation of multidimensional anthropometric, metabolic, and behavioral assessment strategies for earlier identification of hidden cardiometabolic risk.

## Figures and Tables

**Table 1:** Demographic, anthropometric, glycemic, lipid, and vascular characteristics according to adiposity–metabolic discordance phenotypes.

Variable	Concordant Healthy Phenotype	Adiposity-Only Phenotype	Metabolic-Only Phenotype	Concordant High-Risk Phenotype	*p*-Value	Effect Size
*n* (%)	131,010 (78.9)	16,282 (9.8)	15,354 (9.2)	3362 (2.0)	—	—
Age (years)	36.17 ± 9.45	41.06 ± 9.88	42.71 ± 10.11	44.13 ± 10.35	<0.001	0.062 ^a^
Male sex, *n* (%)	71,845 (54.8)	10,925 (67.1)	9742 (63.4)	2652 (78.9)	<0.001	0.21 ^b^
BMI (kg/m^2^)	22.28 ± 1.69	23.16 ± 1.34	22.91 ± 1.50	23.51 ± 1.17	<0.001	0.041 ^a^
Waist circumference (cm)	74.07 ± 6.76	84.92 ± 5.88	76.31 ± 6.49	86.96 ± 6.09	<0.001	0.228 ^a^
Waist-to-height ratio	0.44 ± 0.03	0.51 ± 0.02	0.45 ± 0.03	0.52 ± 0.03	<0.001	0.384 ^a^
METS-VF	5.01 ± 0.72	6.89 ± 0.81	5.48 ± 0.76	7.34 ± 0.88	<0.001	0.425 ^a^
Systolic BP (mmHg)	113.46 ± 12.82	117.25 ± 13.14	122.05 ± 14.92	127.43 ± 16.15	<0.001	0.055 ^a^
Diastolic BP (mmHg)	68.10 ± 9.10	71.02 ± 9.45	74.63 ± 10.01	76.95 ± 10.40	<0.001	0.058 ^a^

Data are presented as mean ± standard deviation or absolute frequency and percentage. BMI: body mass index; METS-VF: Metabolic Score for Visceral Fat; BP: blood pressure. *p*-values were obtained using one-way ANOVA for continuous variables and chi-square tests for categorical variables. Effect sizes correspond to eta squared (η^2^) for continuous variables (^a^) and Cramer’s V for categorical variables (^b^).

**Table 2:** Metabolic and vascular profile according to adiposity–metabolic discordance phenotypes.

Variable	Concordant Healthy Phenotype	Adiposity-Only Phenotype	Metabolic-Only Phenotype	Concordant High-Risk Phenotype	*p*-Value	Effect Size (η^2^)
Fasting glucose (mg/dL)	82.61 ± 10.60	84.95 ± 10.91	89.82 ± 12.73	91.60 ± 13.96	<0.001	0.047
Total cholesterol (mg/dL)	181.54 ± 31.88	188.73 ± 33.15	196.84 ± 35.27	203.42 ± 36.81	<0.001	0.028
LDL cholesterol (mg/dL)	108.72 ± 27.45	114.88 ± 28.31	119.41 ± 30.27	123.92 ± 31.08	<0.001	0.019
HDL cholesterol (mg/dL)	54.87 ± 6.89	53.80 ± 6.34	47.79 ± 7.51	47.68 ± 7.11	<0.001	0.095
Triglycerides (mg/dL)	76.81 ± 28.15	81.17 ± 29.50	171.28 ± 95.88	182.70 ± 96.51	<0.001	0.346
TG/HDL-c ratio	1.42 ± 0.51	1.54 ± 0.58	3.76 ± 1.84	4.03 ± 1.97	<0.001	0.474
TyG index	7.99 ± 0.40	8.06 ± 0.41	8.79 ± 0.54	8.89 ± 0.51	<0.001	0.277
Systolic BP (mmHg)	113.46 ± 12.82	117.25 ± 13.14	122.05 ± 14.92	127.43 ± 16.15	<0.001	0.055
Diastolic BP (mmHg)	68.10 ± 9.10	71.02 ± 9.45	74.63 ± 10.01	76.95 ± 10.40	<0.001	0.058
METS-VF	5.01 ± 0.72	6.89 ± 0.81	5.48 ± 0.76	7.34 ± 0.88	<0.001	0.425

Data are presented as mean ± standard deviation. LDL: low-density lipoprotein; HDL: high-density lipoprotein; TG/HDL-c: triglyceride-to-HDL cholesterol ratio; BP: blood pressure; METS-VF: Metabolic Score for Visceral Fat; TyG: triglyceride–glucose index. *p*-values were obtained using one-way ANOVA. Effect size corresponds to eta squared (η^2^).

**Table 3:** Lifestyle-related characteristics according to adiposity–metabolic discordance phenotypes.

Variable	Concordant Healthy Phenotype	Adiposity-Only Phenotype	Metabolic-Only Phenotype	Concordant High-Risk Phenotype	*p*-Value	Effect Size
Physically active, *n* (%)	113,193 (86.4)	13,281 (81.6)	2917 (19.0)	535 (15.9)	<0.001	0.48
Sedentary lifestyle, *n* (%)	17,817 (13.6)	3001 (18.4)	12,437 (81.0)	2827 (84.1)	<0.001	0.51
Favorable dietary adherence, *n* (%)	104,022 (79.4)	11,348 (69.7)	4329 (28.2)	609 (18.1)	<0.001	0.44
Unfavorable dietary adherence, *n* (%)	26,988 (20.6)	4934 (30.3)	11,025 (71.8)	2753 (81.9)	<0.001	0.44
Current smoking, *n* (%)	42,579 (32.5)	6548 (40.2)	7734 (50.4)	1779 (52.9)	<0.001	0.19
Non-smoker, *n* (%)	88,431 (67.5)	9734 (59.8)	7620 (49.6)	1583 (47.1)	<0.001	0.19

Data are presented as absolute frequencies and percentages. Physically active was defined as ≥600 MET-min/week, whereas sedentary lifestyle was defined as <600 MET-min/week. Favorable dietary adherence was defined as a MEDAS-14 score ≥ 9, whereas unfavorable dietary adherence was defined as a score < 9. Non-smokers included participants who had never smoked and former smokers who had abstained from smoking for at least 12 months. MET: metabolic equivalent of task; MEDAS-14: 14-item Mediterranean Diet Adherence Screener. *p*-values were obtained using chi-square tests. Effect size corresponds to Cramer’s V.

**Table 4:** Prevalence of adverse cardiometabolic outcomes according to adiposity–metabolic discordance phenotypes.

Variable	Concordant Healthy Phenotype	Adiposity-Only Phenotype	Metabolic-Only Phenotype	Concordant High-Risk Phenotype	*p*-Value	Effect Size
Hypertension, *n* (%)	6682 (5.1)	1237 (7.6)	3470 (22.6)	881 (26.2)	<0.001	0.31
Impaired fasting glucose, *n* (%)	5502 (4.2)	1058 (6.5)	4545 (29.6)	1056 (31.4)	<0.001	0.38
Elevated TG/HDL-c ratio, *n* (%)	4061 (3.1)	782 (4.8)	10,318 (67.2)	2411 (71.7)	<0.001	0.69
Elevated METS-VF, *n* (%)	4978 (3.8)	16,282 (100.0)	1996 (13.0)	3362 (100.0)	<0.001	0.84
WtHR ≥ 0.50, *n* (%)	0 (0.0)	16,282 (100.0)	0 (0.0)	3362 (100.0)	<0.001	0.81

Data are presented as absolute frequencies and percentages. TG/HDL-c: triglyceride-to-high-density lipoprotein cholesterol ratio; METS-VF: Metabolic Score for Visceral Fat; WtHR: waist-to-height ratio. *p*-values were obtained using chi-square tests. Effect size corresponds to Cramer’s V.

**Table 5:** ROC curve analysis for discrimination of metabolic dysfunction.

Index	AUC (95% CI)	*p*-Value	Optimal Cut-Off	Sensitivity (%)	Specificity (%)
BMI	0.612 (0.608–0.616)	<0.001	22.60 kg/m^2^	67.2	49.6
Waist circumference	0.590 (0.586–0.594)	<0.001	76.0 cm	63.8	49.9
WtHR	0.594 (0.590–0.599)	<0.001	0.44	81.3	31.9
METS-VF	0.673 (0.669–0.677)	<0.001	5.78	60.7	64.6

AUC: area under the receiver operating characteristic curve; CI: confidence interval; BMI: body mass index; WtHR: waist-to-height ratio; METS-VF: Metabolic Score for Visceral Fat. Metabolic dysfunction was defined as the presence of ≥2 of the five prespecified cardiometabolic criteria. Optimal cut-offs were determined using the Youden index.

**Table 6:** Crude and adjusted logistic regression analyses for adiposity–metabolic discordance phenotypes.

Variable	Adiposity-Only Crude OR (95% CI)	Adiposity-Only Adjusted OR (95% CI)	Metabolic-Only Crude OR (95% CI)	Metabolic-Only Adjusted OR (95% CI)	Concordant High-Risk Crude OR (95% CI)	Concordant High-Risk Adjusted OR (95% CI)
Age (per year increase)	1.01 (1.01–1.01)	1.01 (1.00–1.01)	1.06 (1.06–1.07)	1.03 (1.03–1.03)	1.08 (1.08–1.09)	1.04 (1.04–1.05)
Male sex	4.31 (4.15–4.48)	3.84 (3.69–4.00)	2.15 (2.08–2.23)	2.70 (2.59–2.82)	6.16 (5.63–6.74)	7.65 (6.94–8.43)
BMI (per kg/m^2^ increase)	1.44 (1.42–1.45)	1.31 (1.29–1.32)	1.28 (1.26–1.29)	1.11 (1.10–1.13)	1.75 (1.70–1.80)	1.47 (1.43–1.52)
Sedentary lifestyle	1.44 (1.38–1.50)	1.82 (1.71–1.94)	27.09 (25.94–28.29)	20.40 (19.04–21.85)	33.54 (30.54–36.84)	26.11 (22.49–30.32)
Unfavorable dietary adherence	1.28 (1.23–1.33)	0.89 (0.84–0.94)	13.76 (13.22–14.33)	1.33 (1.24–1.42)	17.47 (15.98–19.09)	1.30 (1.13–1.50)
Current smoking	1.00 (0.97–1.04)	1.01 (0.98–1.05)	1.65 (1.59–1.70)	1.61 (1.55–1.68)	1.82 (1.70–1.95)	1.89 (1.75–2.05)
Model adequacy—adjusted models						
Nagelkerke R^2^	—	0.126	—	0.430	—	0.427
AUC (95% CI)	—	0.727 (0.723–0.732)	—	0.885 (0.882–0.889)	—	0.938 (0.932–0.944)
Hosmer–Lemeshow χ^2^ (df = 8)	—	491.11	—	254.18	—	56.50
Hosmer–Lemeshow *p*-value	—	<0.001	—	<0.001	—	<0.001

Crude and adjusted odds ratios (ORs) with 95% confidence intervals (95% CI) are presented for each phenotype comparison. Crude ORs were obtained from univariable logistic regression models. Adjusted ORs were obtained from multivariable logistic regression models simultaneously including age, sex, BMI, sedentary lifestyle, unfavorable dietary adherence, and current smoking. The concordant healthy phenotype was used as the reference category. Model adequacy for the adjusted models was assessed using Nagelkerke’s pseudo-R^2^, the area under the receiver operating characteristic curve (AUC), and the Hosmer–Lemeshow goodness-of-fit test. BMI: body mass index; AUC: area under the receiver operating characteristic curve; CI: confidence interval.

## Data Availability

The original contributions presented in this study are included in the article/Supplementary Materials. Further inquiries can be directed to the corresponding author.

## Supplementary Materials

The following supporting information can be downloaded at: https://www.mdpi.com/article/10.3390/medsci14050557/s1, Table S1. Sex-stratified-adjusted logistic regression analyses for adiposity–metabolic discordance phenotypes.
