# Supplementary material for: Adiposity–Metabolic Discordance Identifies Cardiometabolically Heterogeneous Profiles in Normal-Weight Adults: A Cross-Sectional Study
Source: Med Sci (Basel). 2026 Sep 9;14(5):557. doi: 10.3390/medsci14050557 (PMC13609116; doi:10.3390/medsci14050557)
Supplement: Supplementary file 1 [file medsci-14-00557-s001.zip › medsci-4530823-supplementary.pdf]

medical sciences

MDPI

Article

Adiposity–Metabolic Discordance Identifies Cardiometabolically Heterogeneous Profiles in Normal-Weight Adults: A Cross-Sectional Study

Table S1. Sex-stratified-adjusted logistic regression analyses for adiposity–metabolic discordance phenotypes.

Variable	Men Adiposity-only OR (95% CI)	Men Metabolic-only OR (95% CI)	Men High-risk OR (95% CI)	Women Adiposity-only OR (95% CI)	Women Metabolic-only OR (95% CI)	Women High-risk OR (95% CI)
Age, per year	1.01 (1.01–1.01)	1.03 (1.02–1.03)	1.04 (1.04–1.05)	1.00 (0.99–1.00)	1.04 (1.03–1.04)	1.03 (1.02–1.04)
BMI, per kg/m²	1.27 (1.25–1.29)	1.10 (1.07–1.12)	1.39 (1.34–1.43)	1.40 (1.37–1.43)	1.13 (1.11–1.16)	1.85 (1.72–1.98)
Sedentary lifestyle	1.46 (1.35–1.58)	28.74 (26.07–31.69)	30.20 (25.48–35.81)	2.20 (1.98–2.44)	12.03 (10.92–13.25)	15.61 (10.94–22.27)
Unfavorable dietary adherence	0.76 (0.71–0.81)	1.47 (1.33–1.63)	1.09 (0.92–1.29)	1.42 (1.28–1.57)	1.17 (1.07–1.29)	2.31 (1.67–3.19)
Current smoking	1.01 (0.97–1.06)	1.68 (1.59–1.78)	1.83 (1.67–2.00)	1.01 (0.94–1.08)	1.51 (1.42–1.60)	2.03 (1.71–2.40)

Odds ratios (ORs) and 95% confidence intervals (95% CI) were obtained from sex-stratified multivariable logistic regression models including age, BMI, sedentary lifestyle, unfavorable dietary adherence, and current smoking. The concordant healthy phenotype was used as the reference category within each sex stratum. BMI: body mass index; CI: confidence interval.

 https://doi.org/10.3390/medsci14050557
